# Developing a peer-led intervention to promote COVID-19 testing in low-income housing settings

**DOI:** 10.3389/fpubh.2023.1096246

**Published:** 2023-05-05

**Authors:** Andrew D. Plunk, Kapri Hannon, Alexandra Carver, Diane Cooper, Debra Grant, Sudie Greene, Emma Morgan, Sarah Gehlert

**Affiliations:** ^1^Department of Pediatrics, Eastern Virginia Medical School, Norfolk, VA, United States; ^2^Housing Collaborative Community Advisory Board, Norfolk, VA, United States; ^3^Brown School of Social Work, Washington University, St. Louis, MO, United States

**Keywords:** COVID-19, community-based participatory research, public housing, distrust, testing

## Abstract

**Background:**

The Housing Collaborative project at Eastern Virginia Medical School has developed a method of adapting public health guidance from public housing communities, which face tremendous health challenges in cardiometabolic health, cancer, and other major health conditions. In this paper, we describe how academic and community partners in the Housing Collaborative came together to do this work with a focus on COVID-19 testing in the context of the emerging pandemic.

**Methods:**

The academic team used virtual community engagement methods to interact with the Housing Collaborative Community Advisory Board (HCCAB) and a separate cohort of research participants (*N* = 102) recruited into a study of distrust in COVID-19 guidance. We conducted a series of 44 focus group interviews with participants on related topics. Results from these interviews were discussed with the HCCAB. We used the collaborative intervention planning framework to inform adaptation of public health guidance on COVID-19 testing delivered in low-income housing settings by including all relevant perspectives.

**Results:**

Participants reported several important barriers to COVID-19 testing related to distrust in the tests and those administering them. Distrust in housing authorities and how they might misuse positive test results seemed to further undermine decision making about COVID-19 testing. Pain associated with testing was also a concern. To address these concerns, a peer-led testing intervention was proposed by the Housing Collaborative. A second round of focus group interviews was then conducted, in which participants reported their approval of the proposed intervention.

**Conclusion:**

Although the COVID-19 pandemic was not our initial focus, we were able to identify a number of barriers to COVID-19 testing in low-income housing settings that can be addressed with adapted public health guidance. We struck a balance between community input and scientific rigor and obtained high quality, honest feedback to inform evidence-based recommendations to guide decisions about health.

## 1. Introduction

Although the importance of including community voices in research has been acknowledged since the mid-1990s (1) and reinforced over time through the development and ongoing operations of the NIH-funded Clinical and Translational Science Centers, how exactly to ensure that these voices break through the dominance of traditional biomedical research in science remains elusive. Institutional barriers to effective community-based participatory research (CBPR) are well-documented and have been noted for decades (2, 3). Improvement occurs in two main ways: through the ability of community engagement to facilitate the translation of biomedical and clinical research into communities and through its ability to inform research about community values and priorities and ameliorate distrust.

Effective CBPR relies on bidirectional communication that is balanced on its ends. Through trial and error, the Housing Collaborative project at Eastern Virginia Medical School (EVMS) has developed a method of establishing public health guidance from a community with tremendous health challenges in cardiometabolic health, cancer, and other major health conditions. In this case, the goal of the Housing Collaborative COVID-19 study was to increase the effectiveness of COVID-19 outreach and guidance in low-income housing communities through a peer-led intervention by Housing Collaborative members. The following article outlines one example of the use of this method to achieve the study goal by outlining the development of a peer-led intervention to support increased at-home COVID-19 testing.

Rather than viewing biomedical and clinical research as scientific, and community engaged research as ascientific, we have applied established principles of intervention research to further our goal of balanced bidirectional communication. Our approach builds on an existing body of peer-led interventions to consider the value of sustainable ties with community members in addressing jointly identified obstacles to health. We believe that our approach to working with communities in which balanced bidirectional communication extends over time can add to the knowledge base on what promotes positive change. We argue that extended communication on a variety of salient topics is essential to closing the gaps between biomedical research and clinical medicine and population health.

### 1.1. Brief overview of peer-led interventions in health

Peer-led interventions extend community-based participatory research to highlight the expertise of community members by including them in conducting interventions to improve community health (4). They can range from those in which peers primarily are involved in delivering interventions to those in which academic and community members work in partnership throughout the research process and across specific projects (5–7). Ross et al. noted in 2010 the need for trust to develop over time so that an environment is created in which each partner is willing to make temporary concessions to produce a long-term collaborative relationship [(4), pp. 2–3].

To date, peer-led interventions have been applied in a variety of arenas and settings, from increasing empathy and self-efficacy among medical students to training peers to provide one-on-one services to persons with serious mental illness (5, 6). Results of these interventions, often measured pre- and post-intervention, largely have been favorable. In the medical student intervention, for example, empathy scores increased despite no change in mental health stigma. In a review of 153 peer-led interventions to promote health and well-being in retirement living, the authors concluded from the seven articles meeting inclusionary criteria that “future studies are needed to better understand how to sustain promising interventions” [(8), p. 11557]. While the low-cost, feasibility, and general favorable outcomes of peer-led interventions have been noted, there is concern about the long-term sustainability of interventions that produce favorable outcomes in testing.

Emerging research involving peer-led support interventions in response to the COVID-19 pandemic has highlighted the importance of recognizing how different definitions of health can have a dramatic influence (9). These include biomedical, relational, and socio-political framings. Biomedical models emphasize disease progression or symptom control, typically outside of social context, which can be a major limitation, as has been highlighted by the experiences of marginalized groups with COVID-19 (10). While relational models recognize social context (11), framing peer interventions solely through a relational lens could fail to appreciate how within-group variation in social norms and a lack of community cohesion could lead to reduced benefit for individuals who might be disempowered relative to the rest of their community (9). Socio-political framings recognize the role that inequalities, disadvantage, and discrimination play in access to services and health outcomes and stress the importance of community-led responses. However, these efforts can be limited when individuals from marginalized groups bear the brunt of the burden for their support. Combining the three perspectives, however, shows promise for creating traction and longevity for peer-led intervention work; in fact, this type of framework appears to be a preferred structure for support by such funders as the Robert Wood Johnson Foundation, given a growing recognition that initiatives will have limited success unless they feature capacity-building and are culturally tailored (12).

### 1.2. COVID-19 testing

Rapid, at-home testing is an important non-pharmaceutical intervention for COVID-19 (13). Research shows that disparities in rapid, at-home COVID-19 testing exist. In a non-probability sample of adults conducted from August 23, 2021 through March 12, 2022 (*N* = 359,399), respondents who used home COVID-19 tests were more likely to report higher incomes, higher educational attainment, and White race. For example, only 2.8% of respondents identifying as Black had used an at-home rapid test in the prior 30 days, compared to 5.9% of White respondents. The authors noted disparities in COVID-19 testing and suggested that additional studies are needed to better understand barriers to testing so that interventions can be developed (14).

While there have been multiple outreach interventions promoting clinic-based testing [e.g., (15)], few published studies have been aimed at overcoming barriers to at-home testing, and existing work might not be well-suited to addressing individual concerns and barriers. For example, the Say Yes! COVID Test campaign employed social marketing techniques in an effort to distribute 66,035 tests in Tennessee and North Carolina communities (14, 16). While this effort is laudable, their primary focus was on promoting increased distribution of tests, rather than overcoming individual-level barriers to testing that might exist after individuals receive their tests. This is an important gap, as our current study highlights.

### 1.3. Housing collaborative at Eastern Virginia Medical School

The Housing Collaborative Community Advisory Board (HCCAB), in partnership with researchers at Eastern Virginia Medical School, was created in 2013 to address the challenges of residents living in public housing in Norfolk, Virginia. The 28 current active members live in some form of low-income housing (e.g., public housing or receive a housing-choice voucher) in one of these Virginia cities: Chesapeake, Hampton, Portsmouth, Newport News, Richmond, Roanoke, Suffolk, and Virginia Beach, in addition to Norfolk (Almost 73,000 low-income housing residents combined live in these cities.) All but two HCCAB members are women and all but one self-identify as Black. The mean age of HCCAB members at the time of this report was 51 years (SD = 15.61). While led by EVMS researchers, faculty members associated with the Housing Collaborative now include co-investigators from several other academic institutions, including Hampton University, Harvard School of Public Health, Norfolk State University, Virginia Commonwealth University and Washington University in St. Louis. The longstanding research partnership spans several grant-funded projects with topics ranging from respiratory health and childhood asthma to studies examining HUD-mandated smoke-free public housing (17–20). Members of the HCCAB contribute to all stages of research, including the development of long-term research agendas and choosing topics covered in individual grant submissions. Monthly in-person meetings were held on the EVMS campus prior to the pandemic, with approximately 15 CAB members in attendance before March 2020. The group shifted to virtual engagement when restrictions on face-to-face interaction were put into place. The HCCAB grew rapidly and transitioned to weekly meetings and a regional presence, with consistently high attendance; this expansion was likely facilitated by the ease of virtual participation and the fact that members were confined to their homes.

The COVID-19 pandemic became the focus of HCCAB discussions in 2020. The group’s weekly reflection focused increasingly on life changes required by the pandemic, including members’ reactions to pandemic-related public health guidance from national and local sources. The academic research team was struck by the candor of the HCCAB as a debate arose nationally about the wisdom and necessity of mandates like vaccination and masking. It became clear that dialogs on community attitudes about COVID-19 precautions were being driven by the broader issue of trust in science. The academic team was able to observe, based on ongoing discussions with the HCCAB, how the group’s trust in science and faith in recommendations changed by virtue of their ongoing relationships with one another and with the research staff members.

### 1.4. Housing collaborative COVID-19 study design

This article describes the process of community-informed adaptation that was part of a study funded by the National Institutes of Health through the Rapid Acceleration of Diagnostics in Underserved Populations (RADx-UP) initiative. Engagement with the HCCAB early in the pandemic suggested that widespread distrust of information about COVID-19, especially when received from public housing authorities, was contributing to low adherence with public health guidance. In particular, the HCCAB had described how recommendations for COVID-19 testing were met with skepticism and suspicion in their communities, a situation that directly contributed to the design of the Housing Collaborative COVID-19 study and demonstrated the importance of increasing the effectiveness of COVID-19 outreach and guidance in low-income communities. This article describes our work with the HCCAB to overcome distrust in COVID-19 testing after the study was funded. We began by recruiting an additional cohort of low-income housing resident research participants with whom we would engage in focus group interviews to examine systematically the phenomena described by the HCCAB. These focus group interviews were analyzed and findings were taken back to the HCCAB to generate discussion on how best to respond to community-identified concerns. We viewed this process, the work of making COVID-19 testing guidance more responsive to community needs, to be intervention adaptation. This was informed by the collaborative intervention planning framework, which applies community-based participatory research principles by fostering joint, balanced conversations between researchers and community members. This process yielded several recommendations, including the articulation of a peer-led COVID-19 testing intervention, on which we sought additional community feedback in another series of focus group discussions.

## 2. Methods

### 2.1. Participant recruitment and support

The Housing Collaborative COVID-19 study was conducted virtually, using digital access capacity provided by the team. Required as part of COVID precautions, digital access actually fostered consistent attendance. Members of the HCCAB and research participants were provided with tablets with high definition webcam, unlimited data connectivity, and, most importantly, ongoing technical support should they experience any problems while participating in study activities. A detailed description of our digital access capacity-building method, which was developed to ensure that engagement with the HCCAB would not be interrupted by the pandemic, is available elsewhere (17).

Before the pandemic, our process for recruiting for the HCCAB began by relationship-building and with sharing project goals and intentions with community members. Restrictions on face-to-face contact required that we begin by asking for referrals from housing authority staff and existing HCCAB members. As we expanded, we also recruited residents using mailers and flyers posted in apartment buildings. Interested individuals were contacted by a research staff member, who provided information about project goals, topics of discussion, HCCAB member responsibilities, and incentives for participating. All HCCAB members received the tablet computer with internet and $10 per hour for every meeting attended.

We used our digital access capacity-building method to recruit a cohort of participants for the Housing Collaborative COVID-19 study beginning in May 2021. Eligibility criteria were being an adult resident of low-income housing in one of the cities listed above. Recruitment was conducted using flyers, re-contact based on participation in previous studies, and referral from other participants and HCCAB members. The cohort has participated in a range of study activities using the provided digital access capacity, including quantitative and qualitative assessment. Participants were offered up to four study activities per month, one of which was a focus group discussion; however, participants were under no obligation to complete any particular activity or to attend specific focus group discussions if they preferred not to participate. As compensation, participants received unlimited data connectivity via provided tablets and $5 per completed research activity, equaling an upper range of $380. Approval was obtained from the EVMS IRB (20-04-NH-0099, 21-03-EX-0069, and 21-03-FB-0046). In total, 84 online focus groups were conducted with 102 participants from June 2021 through September 2022, with the cohort being sampled separately for each topic.

### 2.2. Data collection

This article involves a subset of our data collected during 44 focus group discussions, including 22 discussions on trust in COVID-19 guidance (*n* = 102 participants, with an average of 4.8 attendees per group), 19 discussions on comfort with technology (*n* = 81 participants, with an average of 4.3 participants per group); there were three additional focus group discussions specifically on the proposed peer-led testing intervention (*n* = 13 participants, with an average of 4.33 participants per group), which occurred after conferring with the HCCAB about feedback from the earlier focus groups. Focus groups were convened online using the teleconferencing platform Zoom. Our attendance target for each of the planned discussions was four to six; in practice, attendance ranged from 2 to 10 participants, with nine having fewer than 4 participants. Each discussion was facilitated by three members of the research staff—one moderator who led the discussion and two others who coordinated with participants, obtained consent, and took observational notes on issues such as hesitation or speed in responding. In addition, they were on hand should a participant need technical support. Video and a redundant audio recording of each session, with consent, were obtained so that those involved could reflect on aspects of the discussions. The discussions followed a semi-structured format based on a discussion guide developed in concert with the HCCAB; this format is open-ended, allowing for the discussion to evolve in response to the conversation. Immediately following each focus group discussion, research staff would debrief and discuss any arising or similar themes, interesting topics that could lead to future discussions, and general remarks about the preparation and facilitation process of the discussions for later planning and evaluation. Staff completed field notes and uploaded the notes, along with the video and audio recordings, to a secure server for storage until needed for data analysis. Recordings of the discussions were professionally transcribed. In total, 1,188 pages of single-spaced transcripts were produced during the 44 discussions analyzed for this article. Research staff produced 237 pages of field notes.

### 2.3. Data analysis

The qualitative analyses presented here are part of a larger effort to develop an understanding of low-income housing residents’ distrust in COVID-related public health guidance using focus group principles (21). Discussions were professionally transcribed and then analyzed using a process in which codes and categories were iteratively created to reconcile emerging concepts (22). The first author (an ethicist and social epidemiologist trained in applying qualitative research methods and experienced conducting community-engaged research in low-income settings) and second author (a master’s-level research staff member experienced in facilitating focus group discussions and coding qualitative data) read each transcript to identify emergent concepts, after which they began an iterative process of identifying and reevaluating codes. Inter-coder agreement was reached by consensus. A third member of the team (the senior author; a social scientist with experience in focus group research and qualitative analysis) was available should consensus not occur. The HyperRESEARCH software was used for data organization. Analytic memo writing was utilized to reflect on and process participant responses. Memo writing was also utilized as a tool to connect participant responses across focus group discussions to track any changes in individual- and social-level processes. This first phase of analysis resulted in a list of concepts that was brought to the HCCAB for review.

### 2.4. Adaptation method

We used the collaborative intervention planning framework to achieve the desired balance between hearing community voices and maintaining scientific rigor [(23); see Table 1]. Our ultimate goal was to inform adaptation of public health guidance on COVID-19 testing delivered in low-income housing settings by including all relevant perspectives. The framework applies community-based participatory research principles to an adaptation process that brings together researchers and community members in a structured and systematic way. We aimed to ground our recommendations for practice and policy guidance about COVID-19 in low-income housing residents’ lived experiences as they emerged in our focus group discussions. HCCAB involvement assisting the academic team members with interpretation was crucial to ensure that recommendations reflected community-identified needs.

**Table 1:** Summary of the collaborative intervention planning framework.

Step	Objectives	Activities	Products
1. Setting the stage	Foster partnership and collaboration Clarify CAB members’ roles and responsibilities Introduce project aims, intervention adaptation process, and intervention	Icebreaker activities, mission statement exercises, and group discussions	Mission statement
2. Problem analysis and needs assessment	Identify community needs Discuss how the intervention may or may not address these needs Identify areas for intervention adaptations	Brainstorming exercises, group discussions, development of a logic model, needs assessment	Logic model and needs assessment findings
3. Review of intervention objectives and theoretical foundations	Review the objectives, methods, materials, and theoretical foundations of the intervention Identify specific adaptation to intervention content or delivery	Group discussions and review of intervention components, change objective tables, and intervention’s logic model	Revised logic model and change objective tables of adapted intervention
4. Development of intervention adaptations	Incorporate adaptations into the intervention manual and materials Finalize adapted intervention	Review of intervention manual and materials and group discussions	Intervention manual and materials and training curriculum

Adapted from Cabassa et al. (23).

One hundred forty-one online CAB meetings occurred between March 2020 through October 2022. Of those, 12 meetings were devoted to this adaptation process. On average, 85% of the HCCAB was in attendance at these meetings.

## 3. Results

### 3.1. Focus group feedback regarding barriers to testing

Our research participant cohort consisted primarily of Black members (93%), followed by white members (5%), and a bi- or multi-racial/ethnic identity (2%). Members of the cohort primarily identified as woman/female (74%), followed by “none of these describe me” (14%), man/male (11%), and “prefer not to say” (2%). Age ranged from 18–75 years with a mean age of 53 years (SD = 15.23). Three primary themes and two subthemes emerged from our analysis of focus group feedback.

#### 3.1.1. Theme 1: distrust in COVID-19 testing

Participants reported low trust in COVID-19 testing, which likely affected the decision to seek out a test. This theme was shaped by feedback reflecting low trust in COVID-19 test results coupled with misinformation about the tests themselves.

##### 3.1.1.1. Subtheme 1: distrust of test results compounded by misunderstanding processes

Participants described concern for the motives of the institutions administering and reporting test results [e.g., “I think that test is rigged” (57-year-old Black man)] and the accuracy of the tests. Many of these concerns about test results seemed to be driven by participant confusion about the process of COVID-19 testing, which no one had addressed with them. For example:

*But if you're just testing people and finding it in their blood, why you gotta stick the longest Q-tip up my nose? That's a flu test that you giving. You understand? Like, don't, you giving me a flu test for something that you said that could kill me. You should draw my blood and check and make sure that it ain't already infecting me and it ain't full-blown or I just think the process that they took alone lets you know that it was a bunch of trash behind it in the beginning* [31-year-old Black man].

“That’s a flu test” was a common refrain, mentioned in nine focus group discussions. Relatedly, participants cited confusion about how COVID-19 occurs, when tests are able to detect infection, and how test results might change over time as contributing to their distrust. For example, a participant described how he felt when hearing that someone could get a positive result after testing negative the prior week:

*For me, I didn't see that they were very accurate because in some instances, you would go one place and get the test and they will say, you know, you have to wait a week or 10 days before you get the results. And then you get the results and they say you're negative, but then if you go somewhere else and get the test, then they say you're positive. It was just too much confusion for me* [55-year-old Black man].

While the administration of a COVID-19 test is relatively straightforward, the progression of the disease and what that means for the process of testing and the accuracy of test results can be complicated.

##### 3.1.1.2. Subtheme 2: distrust in institutions leading to COVID-19 testing misinformation

Feedback from roughly one-third of participants (30%) suggested that distrust in institutions providing or promoting testing primed them to be receptive to misinformation. Oftentimes the source of this distrust was the federal government. For example:

*I've seen that the left hand never knows what the right hand is doing. So, on one hand, you may have Dr. Fauci telling you one thing, but then you had Trump saying something totally different, and then you had somebody else saying something totally different from what both of them were saying. So when it comes down to a test that's issued by the government, I'm always going to be skeptic, I'm always going to have my doubts. I'm going to do my own research and I'm going to figure it out for myself* [55-year-old Black man].

It was also common for participants to assume that healthcare institutions had a monetary incentive to report positive cases and treat more COVID-19 patients. This concern was raised in half of the focus group discussions and is described in the following quote:

*I think the results are all misled. I’ve heard the doctors are saying that they are being told to say the test results are valid where they have it and it’s not true. And I heard that a lot of the hospitals are getting money for having a certain amount of people with the COVID. So I think that the testing are all flawed. I think they’re gonna say you got it regardless, if they need a certain percentage of people to have it* [39-year-old Black woman].

Notably, several participants reported not wanting to take tests due to their impression that testing would lead to infection. In justifying this impression, they said those individuals getting tests often ended up having COVID-19. For example, a 59-year-old Black woman participant stated, “I do not know if I would a took that test for the simple fact that a lot that’s getting the test is ending up with the COVID. You see what I’m saying?” This feedback highlights how information is processed in the absence of trust. If one starts with a firm belief that testing is not being done to help those being tested, then it is reasonable to assume that a causal association exists between testing and contracting COVID-19.

#### 3.1.2. Theme 2: fear of pain or discomfort associated with COVID-19 testing

Participants often reported anxiety about testing due to anticipated pain or discomfort. Some participants reported that they had overcome their fears, as in the case of this 58-year-old Black woman who stated, “I was scared for a while. That’s what took me so long. Because people told me it was painful.” Of those who do choose to overcome testing-related anxiety, the need for a test before an upcoming medical procedure was a commonly cited motivation. For example:

*I've not had it. I'm getting ready to have a procedure next month and a day or two before that procedure, I have to have that test. And that is the only thing that's stressing me right now, is that I really don't want them sticking that long Q-tip up my nose* [71-year-old Black woman].

Others opted never to get tested because of what they had been told by others, which seems to have contributed to testing-related misinformation. A 63-year-old Black woman relayed that “I heard different stories, when people took the test, that they stuck it too far up the nose. One lady had to go to the emergency room because he went too far up. So, I never had that done to me.” A 28-year-old Black woman participant reported similar concerns, saying that she had read an article that described how “some people went so far up people nose that like they would hit their brain line, like it would start leaking.”

Several participants also described how educational campaigns promoting testing had contributed to their fear. A 39-year-old White woman stated, “When I first heard about it, I had a flyer and it showed a picture of that whole procedure and I was skeptical. They had their head tilted back and it showed the thing going in the nose and it tells you how deep it goes in. It was just too much.”

#### 3.1.3. Theme 3: concerns about housing undermined the importance of testing

Participants in all focus groups expressed the fear that testing might jeopardize their housing status if housing authority administrators learned of a positive test result. As described by a 70-year-old Black man, “they’d probably put you in quarantine, and try to find a way to get you out of the building.” Many participants seemed to assume that a positive test result would be used against residents who were disliked by staff. For example:

*I don't think it would be a good thing. A lot of times, you can already tell, just from the other questions that you ask them, you can already tell how they feel as far as their bias and their favoritism. So, I don't see that being a good thing, um, or anything that would go in your favor* [55-year-old Black man].

Other feedback seemed to characterize the relationship with the housing authority as fundamentally adversarial. A 70-yer-old Black woman reported the following:

I don't trust them and they may use the information to terminate your lease. They wouldn't say that that was the reason, but they would find a way. I believe they would find a way to terminate your lease. It's ways that you can terminate a lease other than what they have in our contract. But if you don't know that and they come up with these other reasons, then, you know, you, if you don't know, they can take advantage of your lack of knowledge. But I read everything, and anything that looks like a loophole to me, I use it against them.

A perceived lack of confidentiality appeared to compound concerns about privacy. For example, a 70-year-old Black man was concerned that residents in his building would know if he became sick, saying “So if I did have it and went to the hospital, and when I come back, I’m pretty sure everybody in the building would know I had it, and do not go near them. Do not go near them, they have got it.”

### 3.2. Adaptation of COVID-19 testing outreach with the housing collaborative community advisory board

We set aside one meeting for the first step of the process outlined in Table 1. This step was abbreviated given that our partnership with the HCCAB was in place and we had already developed a mission statement guiding our overall work (“To apply our *community awareness* and shared knowledge through networking to *build trust* in COVID-19 guidance, reduce the severity and spread, and *save lives* in our communities”). Our product from the first meeting was an agreement for us to adapt COVID-19 testing guidance in low-income housing settings with an outline of next steps. Five meetings were devoted to problem analysis and needs assessment, primarily using focus group feedback as a guide. While the objectives and theoretical foundations of COVID-19 guidance were ongoing topics of discussion with the HCCAB, we devoted three meetings specifically to exploring these concepts as they related to increasing the effectiveness of COVID-19 testing outreach. An additional three meetings were devoted to the development of intervention adaptation.

The HCCAB recommended three targeted areas of adaptation to increase the perceived usefulness and efficacy of COVID-19 testing:

• Public housing residents would benefit from convenient testing that would not be perceived as linked to the housing authority or another distrusted institution.

HCCAB feedback stressed the importance of convenience while also acknowledging that community-placed testing could easily be perceived as being linked to the housing authority. The HCCAB recommended a community-driven effort to overcome concerns about information being misused by housing authority staff and administration.

• Other residents could benefit from being engaged in a way that mirrored the experience of the Housing Collaborative Community Advisory Board.

Roughly half of HCCAB members exhibited a great deal of distrust in the U.S. pandemic response in 2020. Yet, several HCCAB members described how being authentically engaged with the project about COVID-19 testing and vaccination gradually led them to change their minds. Importantly, this was the case despite a lack of any direct effort by the academic team. They stressed that relationship-building and being treated respectfully were more important than receiving specific content promoting vaccination or testing. When asked what they appreciated about the meetings, HCCAB members variously stated that we “were not pushy,” “were calm,” and “did not act like you are selling something.” HCCAB Members also agreed that getting information from the academic partners on the team and then being able to hear other members’ reactions and reflections helped them develop their own opinions.

• Community members need help addressing their anxiety about the discomfort of COVID-19 testing.

HCCAB members reiterated that unrealistic perceptions about discomfort associated with COVID-19 testing was a real barrier to dealing with the pandemic. They suggested that community members who had undergone COVID-19 testing would likely be best-equipped to help others in their community overcome their anxiety.

Based on these recommendations, the academic team proposed an intervention that would be delivered to community members by HCCAB members serving as peer mentors. Features of the proposed intervention included (1) online delivery using the Zoom platform; (2) a relationship-focused approach, with a majority of the interaction devoted to developing rapport, rather than simply targeting COVID-19 testing; and (3) a peer-mentor demonstration of how to correctly self-administer an at-home COVID-19 test. The HCCAB approved the proposal. Materials outlining the intervention and a training curriculum were created as final products of the adaptation process. The intervention was then taken back to the research participant cohort for their input through an additional round of focus group interviews.

### 3.3. Focus group feedback on proposed peer-mentor COVID-19 testing intervention

Participants indicated that although attitudes about self-administered rapid COVID-19 tests were mixed, receiving direct help with them likely would increase comfort with their use. Several participants noted feeling comfortable with the convenience of rapid tests, yet feeling overwhelmed with self-administering one. For example, two participants described how assistance either had helped them with a prior rapid test or had the potential to do so in the future. Their reports follow:

*That was a good thing. I was able to get tested, and not have to wait in long lines. But I'm a little scared, so I had my friend do it for me. I don't know, sticking the thing up your nose is, I think it's a mind-over-matter thing* [35-year-old Black woman].

*Maybe I'm really feeling bad and I said, oh, you know, I could have COVID; then if I have the test, then I would do it. I would try my best to follow the instructions. And then if, of course, somebody shows me how to do it, yeah, I would do it, yeah* [50-year-old Black man].

Participant feedback also suggested that the proposed peer mentor testing model had the potential to help overcome barriers associated with prior negative experiences. A 63-year-old Black woman participant described this in her feedback about rapid tests (with interviewer content included):

Participant: *I'm afraid to use it. I guess because when I first had the test done, I had to go to a drive-through and the lady that did my test, oh my God, it was the worst experience I ever could have had. She took the Q-tip and she stuck it all the way up in my nose until she pulled blood and tears was just rolling down my eyes.*

Interviewer: *So, have you ever done an at-home test?*

Participant: *No, I'm afraid. I have a test here, but I’m afraid.*

Interviewer: *Okay. So, if someone showed you how to properly do it and how to swab yourself, would you feel comfortable doing it then?*

Participant: *I probably would.*

Interviewer: *Okay, and would you prefer if someone did it, like, over Zoom like how I'm doing it now or would it be better if someone showed you in person?*

Participant: *Ah, the Zoom like we’re doing would be good. The Zoom would be good, yes.*

Overall, feedback was positive about potential help with administering an at-home COVID-19 test delivered by a member of the same community. Notably, no participants were critical of the proposed intervention.

## 4. Discussion

Our goal was to strike a balance between community input and scientific rigor, ultimately to secure community buy-in and obtain high-quality, honest feedback to inform evidence-based recommendations to guide decisions about health. Taken from a broader perspective, we wanted to ensure that communication from communities to investigators was as robust as that from investigators to communities. The process was not intended to be for one project only but rather to establish an ongoing relationship to identify and address community-identified needs in partnership. The onset of the unfolding pandemic required that we communicate virtually with community members about COVID-19. Although not originally planned, this activity resulted in even stronger ongoing participation among group members that will continue as new issues arise.

We were able to identify a number of barriers to COVID-19 testing in public housing settings that can be addressed easily with adapted public health guidance to make outreach more effective and increase testing uptake. The perceived usefulness of testing has likely been undermined by distrust and misunderstanding of the testing process, which seems to be exacerbated by perceptions that COVID-19 testing is painful by design. Misinformation about COVID-19 testing seemed to increase as trust in the test and those administering it declined. We also observed how active distrust in testing could promote conspiratorial thinking (e.g., if testing is assumed not to work but people who get tested develop COVID-19 at higher rates, then those administering tests could be assumed to be somehow causing COVID-19). Given the paucity of research on rapid, at-home COVID-19 testing outreach tailored to address specific community needs, the intervention and the process through which we developed it represent significant steps forward.

With respect to the content of COVID-19 guidance, the trustworthiness of the messenger is likely far more important than the message itself (24). Our interaction with the HCCAB strongly suggests that developing trustworthiness through relationship-building is the primary way to overcome existing distrust. Our proposed peer-led intervention leverages the strength of this approach to address the core barriers raised by residents living in public housing settings.

Our work has several implications for future research. First, the intervention should be piloted to assess whether it increases COVID-19 testing uptake. The relationship building approach can also likely be applied to interventions targeting other health behaviors. Whereas the importance of trust-building is a central theme in the CBPR literature, further research explicitly focusing on relationship building is needed. For example, Jagosh et al. (25) describe “unanticipated benefits” associated with CBPR that primarily work through trust-related mechanisms, including a commitment to power-sharing. Our study suggests that relationship building through CBPR should be considered an intervention in and of itself, particularly in the presence of strong distrust. Researchers should be anticipating these kind of benefits and actively investigating how to promote them.

While a strength of CBPR is that it can be very responsive to community-identified needs, it is important to note that results are often context-specific, which can limit their applicability to other settings. However, we expect findings to remain relevant for low-income housing settings across the U.S., which house a significant number of residents, over 9M, based on 2021 U.S. Department of Housing and Urban Development data (26). Further, our findings could also be generalizable to other marginalized settings characterized by distrust in important institutions.

Although the COVID-19 pandemic was neither the initial nor the sole focus of our efforts, the adaptations and changes that it invoked led to important insights. We used a systematic method to ensure community participation and, in so doing, generated trust. This method is the major contribution of our work that addresses previously identified concerns with the sustainability of peer-led interventions. We also embrace the notion of balancing biomedical, relational, and socio-political aspects of peer support’s impact on health, as described by Mullard et al. (9). In particular, our work offers important practical insights for capturing diverse voices that represent subgroups within marginalized communities. Perhaps the greatest insight is that genuine and ongoing communication will help communities proffer their beliefs and attitudes about important public health issues as it becomes clear that interest in their views is real and valued. The discussion space that is formed becomes an incubator in which genuine interest and sustainable good will can be built and future community health concerns identified and addressed in partnership. We anticipate that strength of the partnerships and openness to participating actively will continue to grow over time.

## Data availability statement

The datasets presented in this article are not readily available because the qualitative data contain many identifiers and complete anonymization is impractical. Requests to access the datasets should be directed to plunkad@evms.edu.

## Ethics statement

The studies involving human participants were reviewed and approved by Eastern Virginia Medical School IRB. The patients/participants provided their written informed consent to participate in this study.

## Author contributions

AP, DG, SuG, and EM designed the research. AP, KH, and AC acquired the data. AP, KH, and SaG analyzed focus group transcript data. AP, KH, AC, DG, and EM took part in the adaptation meetings with the community advisory board. All authors contributed to the article and approved the submitted version.

## Funding

The work was financially supported by the National Cancer Institute of the National Institutes of Health under the award number R37CA245716 (AP, KH, SaG, and AC). The content is solely the responsibility of the authors and does not necessarily represent the official views of the National Institutes of Health.

## Conflict of interest

The authors declare that the research was conducted in the absence of any commercial or financial relationships that could be construed as a potential conflict of interest.

## Publisher’s note

All claims expressed in this article are solely those of the authors and do not necessarily represent those of their affiliated organizations, or those of the publisher, the editors and the reviewers. Any product that may be evaluated in this article, or claim that may be made by its manufacturer, is not guaranteed or endorsed by the publisher.
